# The relationship between vulnerable narcissism and body dissatisfaction among cosplayers: the serial mediating mechanisms of self-objectification and rumination

**DOI:** 10.3389/fpsyg.2026.1822788

**Published:** 2026-05-28

**Authors:** Yu Zhou, Yajuan Huang, Jia Wang, Xin Xu

**Affiliations:** 1 Tianjin Vocational College of Sport, Tianjin, China; 2 School of Education, Tianjin University, Tianjin, China; 3 Institute of Applied Psychology, Tianjin University, Tianjin, China; 4 Laboratory of Suicidal Behavior Research, Tianjin University, Tianjin, China

**Keywords:** body dissatisfaction, cosplayers, rumination, self-objectification, vulnerable narcissism

## Abstract

**Objective:**

This study examined the association between vulnerable narcissism and body dissatisfaction among cosplayers and investigated the serial mediating mechanisms of self-objectification and rumination.

**Methods:**

A total of 335 cosplayers completed validated measures assessing vulnerable narcissism, self-objectification, rumination, and body dissatisfaction using a three-wave survey design. Structural equation modeling and bootstrapped mediation analysis were used to test the hypothetical model.

**Results:**

Vulnerable narcissism, self-objectification, rumination, and body dissatisfaction were significantly and positively intercorrelated (*p* < 0.01). Vulnerable narcissism was positively associated with body dissatisfaction. Self-objectification and rumination independently mediated this relationship. Moreover, a significant serial mediation pathway was identified (vulnerable narcissism → self-objectification → rumination → body dissatisfaction).

**Conclusion:**

Vulnerable narcissism is directly associated with body dissatisfaction among cosplayers and indirectly related to it through both parallel and serial mediation via self-objectification and rumination. Interventions targeting vulnerable narcissistic traits, reducing self-objectification, and disrupting ruminative thought patterns may help alleviate body dissatisfaction in this population.

## 1 Introduction

As physical attractiveness increasingly functions as a form of social capital, body dissatisfaction has emerged as a prevalent psychological concern. Body dissatisfaction refers to negative cognitive and emotional reactions arising from perceived discrepancies between one's actual body and internalized ideals (Dunkley and Brotto, 2021; Sarda et al., 2025). Extensive research has identified physiological (e.g., BMI), psychological (e.g., perfectionism), social (e.g., social media exposure), and cultural (e.g., ethnicity) influences on body dissatisfaction (Muris et al., 2005; Rodgers et al., 2018). Cosplayers represent a distinctive subgroup embedded within digital culture and appearance-oriented communities. China has emerged as the world's largest animation market, with over 580 million pan-2D animation users and more than 120 million core users as of 2025 (China Animation Museum and Zhejiang University, 2025). Cosplayers frequently engage in intensive appearance-related practices, such as corseting or facial contour adjustment, to accurately portray idealized fictional characters (Zhang, 2023). These practices often require heightened body surveillance and may increase vulnerability to body dissatisfaction. Persistent body dissatisfaction has been linked to anxiety, depression, eating disorders, and self-harm (Dunkley and Brotto, 2021; Rodgers et al., 2018). Despite the growing visibility of cosplay culture, limited research has examined psychological risk factors underlying body dissatisfaction in this population. The present study addresses this gap by investigating vulnerable narcissism as a potential predictor and examining the mediating mechanisms of self-objectification and rumination.

### 1.1 Vulnerable narcissism and body dissatisfaction

Vulnerable narcissism is characterized by heightened sensitivity, low self-esteem, insecurity, negative affectivity, and a strong reliance on external validation (Crowe et al., 2019; Miller et al., 2017). Individuals high in vulnerable narcissism tend to internalize shame and anxiety when confronted with negative feedback or unmet interpersonal expectations, which may trigger a range of psychological difficulties (Rogoza et al., 2022). Importantly, vulnerable narcissism may drive individuals to place excessive emphasis on physical appearance and to uphold unrealistically high standards for a “perfect” body. When these standards are not attained, body dissatisfaction may emerge (Purton et al., 2018). Within cosplay culture—where embodying idealized and often exaggerated characters is common—appearance-based comparison and sensitivity to evaluation may be particularly salient. According to the narcissistic spectrum model, individuals high in vulnerable narcissism possess a fragile self-concept contingent on others' approval, which may further intensify concerns about failing to meet socially prescribed appearance norms (Carrotte and Anderson, 2019). Thus, we propose:

***Hypothesis 1:*** Vulnerable narcissism is positively associated with body dissatisfaction among cosplayers.

### 1.2 The mediating mechanism of self-objectification

Self-objectification refers to viewing oneself from an observer's perspective, prioritizing physical appearance over internal attributes (Fredrickson and Roberts, 1997). Objectification theory posits that prolonged exposure to appearance-focused environments increases self-objectification, particularly among individuals embedded in visually evaluative contexts such as cosplay culture. This process manifests in persistent body surveillance and the tendency to treat one's body as an object to be viewed and evaluated primarily on the basis of appearance (Lamp, 2018). Empirical evidence suggests a significant positive association between vulnerable narcissism and elevated levels of self-objectification (Carrotte and Anderson, 2019). Moreover, heightened objectified body consciousness has been linked to adverse psychological outcomes, including the internalization of unrealistic appearance ideals, depressive symptoms, and increased body dissatisfaction (Dakanalis et al., 2015; Saunders et al., 2024). Thus, we propose:

***Hypothesis 2:*** Self-objectification serves as a mediating mechanism in the relationship between vulnerable narcissism and body dissatisfaction among cosplayers.

### 1.3 The mediating mechanism of rumination

Beyond self-objectification, rumination may serve as a crucial cognitive factor explaining the relationship between cosplayers' levels of vulnerable narcissism and body dissatisfaction. Rumination involves repetitive and passive focus on negative emotions and perceived shortcomings (Nolen-Hoeksema, 1991). Individuals high in vulnerable narcissism often experience heightened emotional reactivity and difficulty regulating distress (Loeffler et al., 2020). When faced with perceived appearance inadequacies, they may engage in ruminative thinking, which sustains negative affect and reinforces dissatisfaction. Empirical evidence suggests that rumination strengthens and prolongs body image concerns (Dondzilo et al., 2023). Thus, we propose:

***Hypothesis 3:*** Rumination serves as a mediating mechanism in the relationship between vulnerable narcissism and body dissatisfaction among cosplayers.

### 1.4 The serial mediating mechanism of self-objectification and rumination

Self-objectification among cosplayers also serves as a significant trigger for ruminative thinking. When an individual's level of self-objectification increases, it induces heightened monitoring of one's physical appearance, intensifying body shame and potentially triggering ruminative thoughts (Grabe et al., 2007). This leads cosplayers to allocate more cognitive resources to external physical features, focus more on appearance-related information, and become more attuned to discrepancies between their ideal image and actual self—ultimately triggering negative emotions such as body dissatisfaction (Dondzilo et al., 2023; Fredrickson and Roberts, 1997). Thus, we propose:

***Hypothesis 4:*** Self-objectification and rumination serve as a serial mediating mechanism in the relationship between vulnerable narcissism and body dissatisfaction among cosplayers.

This study aims to investigate the influence of vulnerable narcissism on body dissatisfaction among cosplayers, as well as the serial mediating mechanisms of self-objectification and rumination. By identifying these underlying psychological mechanisms, the present research provides empirical evidence to clarify the etiological factors and cognitive processes associated with body dissatisfaction in this population, thereby offering practical implications for the prevention and intervention of body image concerns among cosplayers.

## 2 Methods

### 2.1 Participants and procedure

The ethics committee of the university with which the corresponding author is affiliated approved this study.

An *a priori* power analysis was conducted using G^*^Power 3.1 (Heinrich Heine University Düsseldorf, Düsseldorf, Germany) (Cohen, 1988). Given the mediation model in the present study, the required sample size was approximated using the multiple linear regression module. Assuming an effect size of *f*^2^ = 0.15, a statistical power of 1 – β = 0.95, and α = 0.05 (two-tailed), the estimated minimum sample size was 115. Subsequently, to mitigate common method variance (CMV), a multi-wave measurement approach was implemented. Data were collected at three time points, each separated by approximately 1 month, using convenience sampling via an online survey platform (https://www.credamo.com). At time 1 (T1), participants reported demographic information and vulnerable narcissism (*n* = 535). At time 2 (T2), they completed measures of self-objectification and rumination (*n* = 370). At time 3 (T3), body dissatisfaction was assessed (*n* = 362). Unique identification codes were used to match responses across waves. After excluding invalid responses based on repeated patterns and abnormal completion times, 335 valid cases were retained (response rate = 62.6%). The final sample included 51 males (15.2%) and 284 females (84.8%), with a mean age of 19.10 years (SD = 2.56).

### 2.2 Measures

#### 2.2.1 Vulnerable narcissism

Vulnerable narcissism was measured using the unidimensional Hypersensitivity Narcissistic Scale (HSNS; Hendin and Cheek, 1997). It includes ten items (e.g., “I often interpret others' comments in a personal way”) evaluated on a five-point scale (1 = Strongly disagree, 5 = Strongly agree). Cronbach's alpha was 0.78 in the current study.

#### 2.2.2 Self-objectification

Self-objectification was assessed using the Body Surveillance subscale of the Objectified Body Consciousness Scale (OBCS; McKinley and Hyde, 1996). The Body Surveillance subscale was selected as it directly measures individuals' habitual attention to body appearance. It includes eight items (e.g., “When I can't control my weight, I feel like something is wrong with me”) evaluated on a seven-point frequency scale (1 = Not at all true, 7 = Completely true). Cronbach's alpha was 0.77 in the current study.

#### 2.2.3 Rumination

Rumination was assessed with the Chinese revised version of the Ruminative Responses Scale (RRS), which was originally developed by Nolen-Hoeksema (1991)) and revised by Han and Yang (2009)). The scale includes three dimensions: symptom rumination (e.g., “I often think about how lonely I am”), reflective pondering (e.g., “I often think about why things are this way”), and obsessive thinking (e.g., “I often think about why I am always like this”). It consists of 22 items, evaluated on a four-point scale (1 = never, 4 = always). Cronbach's alpha was 0.95 in the current study.

#### 2.2.4 Body dissatisfaction

Body dissatisfaction was measured using the four dimensions of the Physical Self Scale (PSS; Liu, 2009): overall dissatisfaction (e.g., “I am proud of my body”), fat dissatisfaction (e.g., “People I like think I'm fat”), appearance dissatisfaction (e.g., “I worry about my looks”), and height dissatisfaction (e.g., “Being short is a major regret of mine”). It includes 27 items, evaluated on a five-point scale (1 = not at all true, 5 = completely true). Cronbach's alpha was 0.91 in the current study.

### 2.3 Statistical analysis

All data were analyzed using SPSS 27 & AMOS 28 (IBM Corp., Armonk, NY, USA). First, descriptive statistics and bivariate correlations were computed using SPSS. Then, structural equation modeling (SEM) with path analysis was conducted in AMOS to test the hypothesized mediation model, allowing for the estimation of relationships among latent variables while accounting for measurement error (Nguyen and Stinglhamber, 2021). Bootstrapping analyses (5,000 resamples) were performed to examine the significance of indirect effects.

## 3 Results

### 3.1 Common method variance analysis

Procedural remedies, including time-lagged data collection and anonymous responses, were implemented to reduce common method variance (Podsakoff et al., 2003). Harman's single-factor test indicated that the largest factor accounted for 24.99% of the total variance, which is below the 40% threshold (Williams et al., 1989). Therefore, common method variance was unlikely to substantially bias the results.

### 3.2 Descriptive statistics and correlation analysis

Bivariate correlations among all variables are shown in Table 1. Positive correlations were observed among vulnerable narcissism, self-objectification, rumination, and body dissatisfaction (*p* < 0.01), thereby providing a basis for subsequent hypothesis testing.

**Table 1:** Means, standard deviations, and zero-order correlations (*N* = 335).

Variable	*M*	SD	1	2	3	4
1. Vulnerable narcissism	33.23	6.34	1			
2. Self-objectification	35.13	8.51	0.25^**^	1		
3. Rumination	49.39	14.24	0.33^**^	0.36^**^	1	
4. Body dissatisfaction	75.87	19.85	0.22^**^	0.37^**^	0.37^**^	1

M, mean; SD, standard deviation; ^**^p < 0.01.

Descriptive analyses were performed, and gender differences were tested applying a Bonferroni correction (see Table 2). The results indicate no statistically significant gender differences in vulnerable narcissism, self-objectification, rumination, or body dissatisfaction (*p* > 0.05). However, given the small male subsample (*n* = 51, 15.2% of the total sample), the statistical power to detect gender differences was limited. Therefore, these non-significant results should not be interpreted as evidence of equivalence between males and females.

**Table 2:** Descriptive statistics and gender comparisons for study variables (*M* ± SD).

Variable	Gender		*t*	*p*
	Male	Female		
Vulnerable narcissism	32.71 ± 5.14	33.32 ± 6.53	−0.64	0.52
Self-objectification	34.80 ± 7.23	35.19 ± 8.73	−0.30	0.76
Rumination	49.88 ± 13.18	49.30 ± 14.44	0.27	0.79
Body dissatisfaction	75.02 ± 19.16	76.03 ± 20.00	−0.33	0.74

df = 333 for all t-tests. Bonferroni correction was applied to account for multiple comparisons, ensuring control of the family-wise error rate.

### 3.3 Hypothesis tests

**Figure 1:**
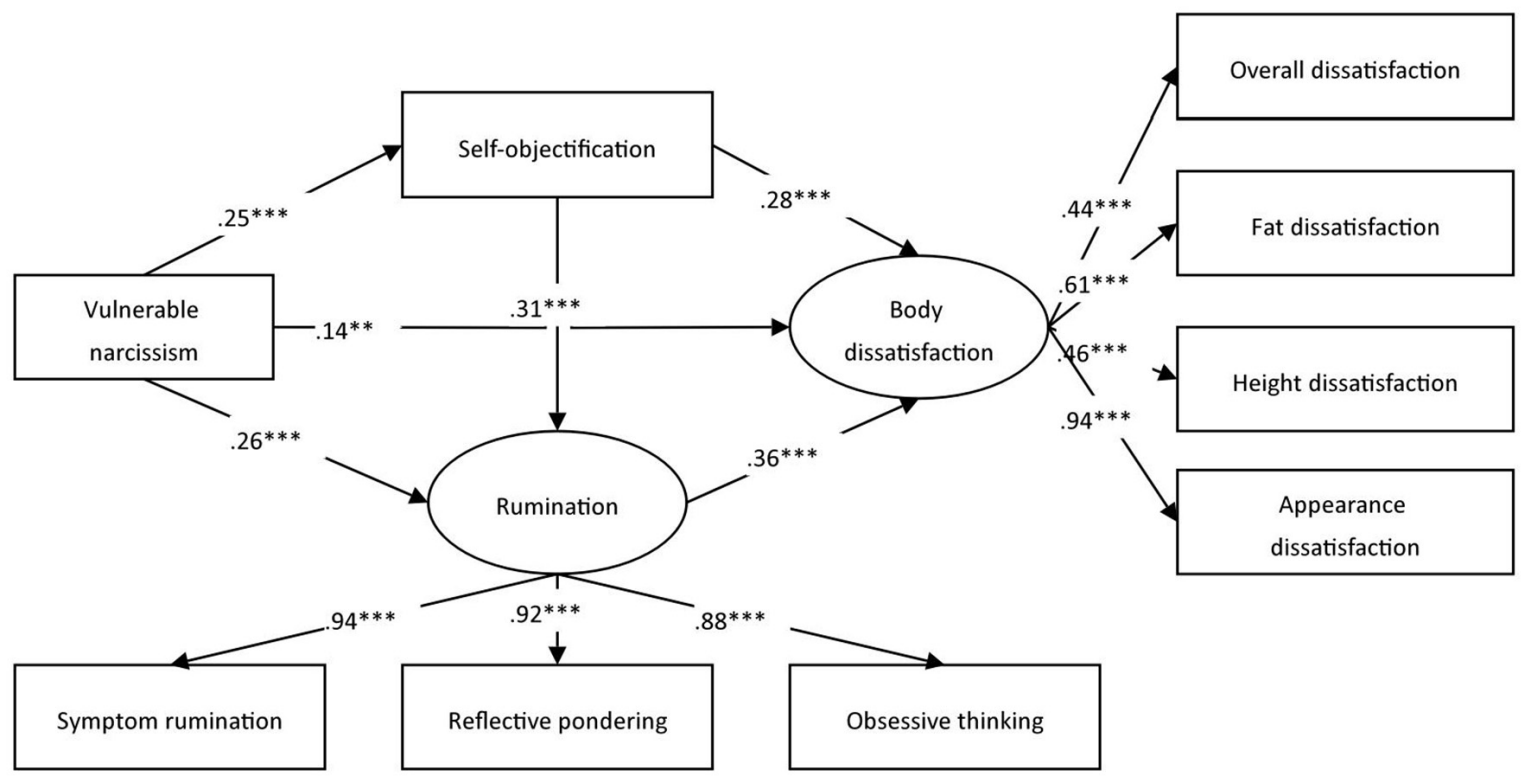
Results of structural equation modeling analyses. **p < 0.01 and ***p < 0.001.

The structural equation model was used to examine the mediating mechanisms of self-objectification and rumination in the effect of vulnerable narcissism on body dissatisfaction. The model showed a good fit to the data (χ^2^*/*d*f* = 1.82, RMSEA = 0.05, CFI = 0.98, IFI = 0.98, GFI = 0.97). The standardized parameter estimates are presented in Figure 1. Vulnerable narcissism was positively associated with self-objectification (β = 0.25, *p* < 0.001) and rumination (β = 0.26, *p* < 0.001). Self-objectification was positively associated with rumination (β = 0.31, *p* < 0.001) and body dissatisfaction (β = 0.28, *p* < 0.001), while rumination was positively associated with body dissatisfaction (β = 0.36, *p* < 0.001). In addition, after self-objectification and rumination were included in the model, the direct effect of vulnerable narcissism on body dissatisfaction remained significant (β = 0.14, *p* < 0.01), indicating a partial mediating mechanism.

The results of the bootstrap analysis (see Table 3) showed that vulnerable narcissism had a significant indirect effect on body dissatisfaction through self-objectification [indirect effect = 0.20, SE = 0.06, 95% CI = (0.09, 0.34)], through rumination [indirect effect = 0.20, SE = 0.06, 95% CI = (0.09, 0.33)], and through the serial pathway of self-objectification and rumination [indirect effect = 0.06, SE = 0.02, 95% CI = (0.02, 0.11)]. The total indirect effect was also significant (indirect effect = 0.46, SE = 0.09, 95% CI = (0.29, 0.66)]. Bootstrapping results indicated that all indirect effects were significant, as the 95% confidence intervals did not include zero. Together with the significant direct effect of vulnerable narcissism on body dissatisfaction, these findings support a partial mediation model.

**Table 3:** Unstandardized indirect and total indirect effects of vulnerable narcissism on body dissatisfaction (*N* = 335).

Model pathways	Effect	Boot SE	95% CI
Vulnerable narcissism → self-objectification → body dissatisfaction	0.20	0.06	[0.09, 0.34]
Vulnerable narcissism → rumination → body dissatisfaction	0.20	0.06	[0.09, 0.33]
Vulnerable narcissism → self-objectification → rumination → body dissatisfaction	0.06	0.02	[0.02, 0.11]
Total indirect effect	0.46	0.09	[0.29,0.66]

Bootstrapping performed with 5,000 resamples; 95% bias-corrected confidence intervals reported. Indirect effects and total effects are unstandardized.

## 4 Discussion

### 4.1 Summary

The findings of this study indicate that vulnerable narcissism was positively associated with body dissatisfaction among cosplayers. More importantly, after self-objectification and rumination were included in the model, the direct effect of vulnerable narcissism on body dissatisfaction remained significant, indicating partial mediation and supporting Hypothesis 1. According to the narcissism spectrum model, individuals high in vulnerable narcissism typically exhibit heightened sensitivity, emotional vulnerability, avoidance tendencies, and unstable self-esteem (Crowe et al., 2019; Miller et al., 2017; Rogoza et al., 2022). They are inclined toward negative self-evaluations and heightened attention to personal shortcomings, which may increase dissatisfaction with their body image. In addition, individuals with vulnerable narcissism often struggle with emotional regulation and lack effective coping strategies, making them more susceptible to negative emotional states such as anxiety and depression. These maladaptive emotional experiences may ultimately translate into increased body dissatisfaction (Loeffler et al., 2020).

Second, the findings demonstrate that vulnerable narcissism is associated with cosplayers' body dissatisfaction through increased self-objectification, indicating a mediating mechanism of self-objectification and supporting Hypothesis 2. According to objectification theory, individuals who habitually monitor themselves from an observer's perspective are more likely to develop self-objectification, which in turn intensifies body dissatisfaction and appearance-related anxiety, and may contribute to eating disorders and depressive symptoms (Tylka et al., 2023). Individuals high in vulnerable narcissism, due to their heightened sensitivity to others' evaluations and strong dependence on external validation, are particularly prone to viewing their bodies from an external standpoint. They tend to internalize societal appearance standards and become acutely aware of how they may be perceived by others (Carrotte and Anderson, 2019; Purton et al., 2018). When they perceive their appearance as falling short of these standards, they are more likely to experience self-criticism, shame, and fluctuations in self-esteem, which may further contribute to body dissatisfaction.

Furthermore, the results reveal that rumination is positively associated with both vulnerable narcissism and body dissatisfaction. Moreover, rumination appears to strengthen the association of vulnerable narcissism on body dissatisfaction, thereby supporting Hypothesis 3. Individuals with vulnerable narcissism are highly sensitive to external evaluations and threats to their self-image. When their physical appearance fails to meet expectations or when they receive negative feedback, they may experience heightened self-doubt and anxiety (Rogoza et al., 2022; Purton et al., 2018). As a maladaptive coping strategy, rumination involves repeatedly focusing on perceived flaws and negative evaluations, leading individuals to internalize external judgments (Feng et al., 2023). This repetitive negative thinking reinforces adverse emotions and hinders effective problem-solving, ultimately contributing to persistent body dissatisfaction and diminished self-worth.

Notably, the findings further indicate that vulnerable narcissism is associated with body dissatisfaction through a serial mediation pathway involving self-objectification and rumination, thereby supporting Hypothesis 4. This result not only highlights the close association between self-objectification and rumination but also clarifies the mechanism through which vulnerable narcissism exerts its influence on body dissatisfaction. Specifically, individuals with fragile narcissistic traits are more likely to internalize society's appearance-focused values, leading to persistent self-monitoring and the instrumentalization of their bodies—core features of self-objectification. During role performance, this heightened self-objectification may encourage frequent upward appearance comparisons, which can activate maladaptive cognitive processes, such as obsessive thoughts about perceived body discrepancies, repetitive rumination on negative emotions, and anticipatory rehearsal of social scenarios. These processes ultimately intensify negative emotional outcomes, including body dissatisfaction and anxiety (Dondzilo et al., 2023).

### 4.2 Theoretical and practical implications

This study offers important theoretical and practical contributions. Theoretically, by integrating the narcissism spectrum model with objectification theory, it proposes a comprehensive explanatory framework of “narcissistic drive–objectification reinforcement–rumination maintenance.” This framework deepens our understanding of the relationship between vulnerable narcissism and body dissatisfaction and provides a foundation for future cross-theoretical integration. Practically, the findings offer valuable implications for mental health interventions targeting cosplayers. Given the prevalence of body dissatisfaction within this population, intervention programs grounded in cognitive-behavioral therapy may be developed to reduce maladaptive self-objectification and rumination patterns, thereby promoting healthier body image and psychological wellbeing.

### 4.3 Limitations and future research

Despite its contributions, this study has several limitations. First, although a three-wave design was employed, the time intervals between measurements were relatively short, which limits strong causal inferences. Future research should adopt longitudinal or experimental designs to better establish causal relationships. Second, the sample was obtained through convenience sampling and was predominantly female (84.8%), with only 15.2% male participants. This substantial gender imbalance limits the generalizability of the findings and reduces the statistical power to detect gender differences. Consequently, conclusions regarding gender comparisons should be drawn with caution. Future studies should aim to include more balanced and representative samples to allow for more reliable gender analyses. Third, all variables were assessed using self-report measures, which may introduce common method bias and social desirability effects. Future research is encouraged to adopt multi-method approaches, such as behavioral observations or informant reports, to enhance methodological rigor.

## 5 Conclusion

Vulnerable narcissism is not only associated with cosplayers' body dissatisfaction through self-objectification and rumination separately, but may also involve a serial mediation mechanism: vulnerable narcissism first relates to increased self-objectification in cosplayers, which then intensifies rumination, ultimately increasing body dissatisfaction.

## Data Availability

The raw data supporting the conclusions of this article will be made available by the authors, without undue reservation.

## References

[B1] Carrotte E. Anderson J. (2019). Risk factor or protective feature? The roles of grandiose and hypersensitive narcissism in explaining the relationship between self-objectification and body image concerns. Sex Roles 80, 458–468. doi: 10.1007/s11199-018-0948-y

[B2] China Animation Museum and Zhejiang University (2025). 2025 China Youth Animation Talent Growth Trends and Future Development Research Report. Available online at: http://www.hhtz.gov.cn/art/2025/10/27/art_1485824_59079339.html (Accessed May 8, 2026).

[B3] Cohen J. (1988). Statistical Power Analysis for the Behavioral Sciences, 2nd Edn. New York, NY: Routledge.

[B4] Crowe M. L. Lynam D. R. Campbell W. K. Miller J. D. (2019). Exploring the structure of narcissism: toward an integrated solution. J. Pers. 87, 1151–1169. doi: 10.1111/jopy.12464 30742713

[B5] Dakanalis A. Carrà G. Calogero R. Fida R. Clerici M. Zanetti M. A. . (2015). The developmental effects of media-ideal internalization and self-objectification processes on adolescents' negative body feelings, dietary restraint, and binge eating. Eur. Child Adolesc. Psychiatry 24, 997–1010. doi: 10.1007/s00787-014-0649-1 25416025

[B6] Dondzilo L. Basanovic J. Grafton B. Bell J. Turnbull G. MacLeod C. (2023). A serial mediation model of attentional engagement with thin bodies on body dissatisfaction: the role of appearance comparisons and rumination. Curr. Psychol. 42, 1896–1904. doi: 10.1007/s12144-021-01574-1

[B7] Dunkley C. R. Brotto L. A. (2021). Disordered eating and body dissatisfaction associated with sexual concerns in undergraduate women. J. Sex. Marital Ther. 47, 460–480. doi: 10.1080/0092623X.2021.1898502 33730967

[B8] Feng Q. S. Zhou Z. K. Sun X. J. Zhang Y. H. Lian S. L. (2023). Negative life events and internalizing problems among junior high school students: the mediating role of rumination and the moderating role of peer attachment. Psychol. Dev. Educ. 39, 419–428.

[B9] Fredrickson B. L. Roberts T. (1997). Objectification theory: toward understanding women's lived experiences and mental health risks. Psychol. Women Q. 21, 173–206. doi: 10.1111/j.1471-6402.1997.tb00108.x

[B10] Grabe S. Hyde J. S. Lindberg S. M. (2007). Body objectification and depression in adolescents: the role of gender, shame, and rumination. Psychol. Women Q. 31, 164–175. doi: 10.1111/j.1471-6402.2007.00350.x

[B11] Han X. Yang H. F. (2009). The Chinese adaptation of the Nolen-Hoeksema ruminative responses scale. Chin. J. Clin. Psychol. 17, 550–551.

[B12] Hendin H. M. Cheek J. M. (1997). Assessing hypersensitive narcissism: a reexamination of Murray's narcissism scale. J. Res. Pers. 31, 588–599. doi: 10.1006/jrpe.1997.2204

[B13] Lamp S. (2018). The Sexy Pikachu Effect: Empowerment and Objectification in Women Who Cosplay (Student Research Submission No. 295), University of Mary Washington.

[B14] Liu D. (2009). The Influence of Mass Media and Peers on College Students' Body Image (Master's thesis). Xiamen University.

[B15] Loeffler L. A. Huebben A. K. Radke S. Habel U. Derntl B. (2020). The association between vulnerable and grandiose narcissism and emotion regulation. Front. Psychol. 11:519330. doi: 10.3389/fpsyg.2020.519330 33178059 PMC7593238

[B16] McKinley N. M. Hyde J. S. (1996). The objectified body consciousness scale: development and validation. Psychol. Women Q. 20, 181–215. doi: 10.1111/j.1471-6402.1996.tb00467.x

[B17] Miller J. D. Lynam D. R. Hyatt C. S. Campbell W. K. (2017). Controversies in narcissism. Annu. Rev. Clin. Psychol. 13, 291–315. doi: 10.1146/annurev-clinpsy-032816-045244 28301765

[B18] Muris P. Meesters C. van de Blom W. Mayer B. (2005). Biological, psychological, and sociocultural correlates of body change strategies and eating problems in adolescent boys and girls. Eat. Behav. 6, 11–22. doi: 10.1016/j.eatbeh.2004.03.002 15567107

[B19] Nguyen N. Stinglhamber F. (2021). Emotional labor and core self-evaluations as mediators between organizational dehumanization and job satisfaction. Curr. Psychol. 40, 831–839. doi: 10.1007/s12144-018-9988-2

[B20] Nolen-Hoeksema S. (1991). Responses to depression and their effects on the duration of depressive episodes. J. Abnorm. Psychol. 100, 569–582. doi: 10.1037/0021-843X.100.4.569 1757671

[B21] Podsakoff P. M. MacKenzie S. B. Lee J. Y. Podsakoff N. P. (2003). Common method biases in behavioral research: a critical review of the literature and recommended remedies. J. Appl. Psychol. 88, 879–903. doi: 10.1037/0021-9010.88.5.879 14516251

[B22] Purton T. Officer C. Bullivant B. Mitchison D. Griffiths S. Murray S. B. . (2018). Body dissatisfaction, narcissism and self-esteem in young men and women: a moderated mediation analysis. Pers. Individ. Dif. 131, 99–104. doi: 10.1016/j.paid.2018.04.010

[B23] Rodgers R. F. O'Flynn J. L. Bourdeau A. Zimmerman E. (2018). A biopsychosocial model of body image, disordered eating, and breastfeeding among postpartum women. Appetite 126, 163–168. doi: 10.1016/j.appet.2018.04.007 29649515

[B24] Rogoza R. Cieciuch J. Strus W. (2022). Vulnerable isolation and enmity concept: disentangling the blue and dark face of vulnerable narcissism. J. Res. Pers. 96:104167. doi: 10.1016/j.jrp.2021.104167

[B25] Sarda E. El-Jor C. Shankland R. Hallez Q. Patiram D. Nguyen C. . (2025). Social media use and roles of self-objectification, self-compassion and body image concerns: a systematic review. J. Eat. Disord. 13:192. doi: 10.1186/s40337-025-01353-4 40877959 PMC12395811

[B26] Saunders J. F. Nutter S. Waugh R. Hayden K. A. (2024). Testing body-related components of objectification theory: a meta-analysis of the relations between body shame, self-objectification, and body dissatisfaction. Body Image 50:101738. doi: 10.1016/j.bodyim.2024.101738 38850716

[B27] Tylka T. L. Rodgers R. F. Calogero R. M. Thompson J. K. Harriger J. A. (2023). Integrating social media variables as predictors, mediators, and moderators within body image frameworks. Body Image 44, 197–221. doi: 10.1016/j.bodyim.2023.01.004 36709634

[B28] Williams L. J. Cote J. A. Buckley M. R. (1989). Lack of method variance in self-reported affect and perceptions of work. J. Appl. Psychol. 74, 462–468. doi: 10.1037/0021-9010.74.3.462

[B29] Zhang Q. Y. (2023). Intertwined self-image and engraved body: a case study of cosplayers' bodily practices. Folk Cult Forum 1, 84–96.

